# Different outcomes of a cardiac rehabilitation programme in functional parameters among myocardial infarction survivors according to ejection fraction

**DOI:** 10.1007/s12471-019-1269-7

**Published:** 2019-04-11

**Authors:** E. M. Vilela, R. Ladeiras-Lopes, C. Ruivo, S. Torres, J. Braga, M. Fonseca, J. Ribeiro, J. Primo, R. Fontes-Carvalho, L. Campos, F. Miranda, J. P. L. Nunes, V. Gama, M. Teixeira, P. Braga

**Affiliations:** 1 Department of Cardiology, Gaia Hospital Centre, Vila Nova de Gaia, Portugal; 2 Department of Cardiology, Leiria Hospital Centre, Leiria, Portugal; 3 Department of Physical and Rehabilitation Medicine, Gaia Hospital Centre, Vila Nova de Gaia, Portugal; 4 Faculty of Medicine, Porto University, Porto, Portugal

**Keywords:** Cardiac rehabilitation, Acute myocardial infarction

## Abstract

**Introduction:**

Exercise-based cardiac rehabilitation (EBCR) is part of the management of patients who have suffered an acute myocardial infarction (AMI). Patients with a reduced ejection fraction (EF) comprise a higher-risk subgroup and are referred less often for these programmes. This study aimed at assessing the impact of the baseline EF on the functional benefits, as assessed by peak oxygen uptake (pVO_2_) and exercise duration, of an EBCR programme in AMI survivors.

**Methods:**

Observational, retrospective cohort study including all patients admitted to a tertiary centre due to an AMI who completed a phase II EBCR programme after discharge, between November 2012 and April 2017. Functional parameters were assessed by a symptom-limited cardiopulmonary exercise test.

**Results:**

A total of 379 patients were included [40.9% with reduced EF (<50%) at discharge]. After the programme, pVO_2_ and exercise duration increased significantly (*p* < 0.001). Patients with a reduced EF had a lower pVO_2_ and completed a shorter duration of exercise at the beginning and end of the programme. This group presented a higher increase in pVO_2_ (*p* = 0.001) and exercise duration (*p* = 0.007). This was maintained after adjusting for age, gender, history of coronary artery disease, number of sessions, Killip classification, arterial hypertension, dyslipidaemia, diabetes mellitus, smoking status and baseline pVO_2_.

**Conclusion:**

A phase II EBCR programme was associated with significant improvements in pVO_2_ and exercise duration among AMI survivors, irrespective of baseline EF classification. Those with a reduced baseline EF derived an even greater improvement, highlighting the importance of EBCR in this subgroup of patients.

## What’s new?

- Acute myocardial infarction survivors with a reduced ejection fraction at discharge have a significantly lower functional capacity, as assessed by cardiopulmonary exercise testing, than those with a preserved ejection fraction.
- This subgroup, however, derives even greater functional benefits from an exercise-based cardiac rehabilitation programme.
- This finding is confirmed even after adjusting for several potential confounders associated with this higher-risk population.

## Introduction

Exercise-based cardiac rehabilitation (EBCR) is an important part of secondary prevention for patients after an acute myocardial infarction (AMI) [1–3]. Several benefits, namely in terms of cardiovascular (CV) endpoints such as mortality and hospitalisations, have been attributed to the participation in these programmes [4, 5]. EBCR exerts several physiological effects by both cardiac and extra-cardiac mechanisms (namely in the peripheral vasculature, pulmonary and musculoskeletal function) [6–10]. These effects range from enhancements in cardiac contractility and chronotropic reserve to anti-inflammatory effects, improved endothelial relaxation and an overall improved aerobic capacity [2, 6, 8].

Cardiopulmonary exercise testing (CPET), being capable of providing functional information relating to several of these components (namely the cardiovascular, respiratory and muscular systems), is an important tool in the global assessment of individuals undergoing EBCR [11, 12]. Importantly, the association between overall functional capacity (sometimes referred to as cardiorespiratory fitness), as assessed by parameters such as the peak oxygen uptake (pVO_2_), and CV events in distinct clinical settings has been thoroughly described [13–16].

Although the importance of EBCR after an AMI is well established, several factors which pertain to its optimal application have not yet been fully ascertained [11, 12, 17]. Importantly, AMI survivors with a reduced ejection fraction (EF) represent a higher-risk subgroup [3] and in some series are referred less often for these programmes [8, 18, 19]. Additionally, the impact of EBCR across different ranges of EF has been the focus of heightened interest [8, 19]. This stems from the fact that, although there are currently numerous parameters of potential interest in the prognostic assessment after an AMI, EF remains the most important parameter in terms of clinical decision-making and overall prognosis [3, 20]. Among the higher-risk subset of patients with a reduced EF, optimising strategies to reduce residual risk are of paramount relevance [2, 7, 19, 21].

This study aimed at assessing the impact of the baseline EF on the benefits of a contemporary EBCR programme, as assessed by pVO_2_ and exercise duration, in AMI survivors.

## Methods

This was an observational, retrospective cohort study. The eligible population comprised all patients who were discharged from the Cardiology Department of the Gaia/Espinho Hospital Centre (a tertiary hospital in Portugal) with the diagnosis of an AMI (according to the International Classification of Diseases, 9th edition), between November 2012 and April 2017.

In order to be included, patients had to have completed a phase II EBCR programme (including at least two assessments in a consultation with a physical medicine specialist and performance of a CPET at the beginning and at the end of the programme).

### EBCR protocol

The EBCR programme consisted of a predefined 8‑week (three sessions per week) outpatient protocol, including endurance and resistance training supervised by a physical therapist. Before starting, patients were clinically assessed and underwent a CPET. Sessions consisted of circa 10 min of warm-up, 50 min of aerobic (continuous) and resistance training, and 10 min of cooldown [7]. Training intensity was individually prescribed by an expert in EBCR to a target heart rate (HR) of 70–85% of the maximal HR achieved in baseline CPET, and fatigue was measured with the Borg scale [7].

### Cardiopulmonary exercise testing

At the beginning and at the end of the programme, patients underwent a symptom-limited CPET on a treadmill (Mortara X‑Scribe, Mortara Instruments, Milwaukee, WI, USA) using either a modification of the Bruce protocol or a variation of this protocol (in highly deconditioned patients).

The following variables were calculated: pVO_2_ measured in millilitres per kilogram per minute (ml/kg/min); peak respiratory exchange ratio (RER), defined by the ratio of CO_2_ production to O_2_ consumption at peak effort; exercise duration expressed in seconds (s). Patients were not asked to discontinue beta-blockers before the test.

### Clinical, analytical and echocardiographic variables

Data were collected for clinical, analytical and echocardiographic variables according to the data present on hospital records (Appendix). Arterial hypertension was defined according to the presence of this diagnosis on clinical files. Dyslipidaemia was defined according to previous diagnosis, or the use of anti-dyslipidaemic medication prior to admission, or having a low-density lipoprotein cholesterol ≥190 mg/dl [1]. Diabetes mellitus was defined according to previous diagnosis, or the use of anti-diabetic agents prior to admission, or having a glycated haemoglobin ≥6.5% [22].

Left ventricular (LV) EF was evaluated by the biplane Simpson’s method, and collected according to pre-discharge assessment. Measurements were performed on one of four ultrasound systems. Echocardiograms were assessed in accordance with the guidelines [23] and validated by an experienced cardiologist. Patients were dichotomised as having preserved (≥50%) or reduced EF (<50%). Although some studies used a lower cut-off value for defining a reduced EF (of 40%) [19, 24], a higher level was used given the data which support the range of 40–50% as being within the scope of LV systolic dysfunction [23] and have an impact on CV events [20].

### Statistical analysis

Continuous variables were expressed as mean ± standard deviation or as median [percentile 25–75, interquartile range (IQR)] according to the normality of the distribution. Categorical variables were expressed as absolute count and percentage. Continuous variables were compared using unpaired or paired *t*-test for those with normal distribution, or with the Mann-Whitney or Wilcoxon tests (for unmatched and matched data, respectively). Categorical variables were compared with the chi-square test. Normality of distribution was assessed using the Kolmogorov-Smirnov test.

Linear regression analysis was used to evaluate if EF was a significant predictor of the change in pVO_2_ irrespective of potential confounders. Age, gender, prior coronary artery disease (CAD), number of sessions, Killip classification, arterial hypertension, dyslipidaemia, diabetes mellitus, smoking status and baseline pVO_2_ were forced into the model. All results were two-sided, and a *p*-value under 0.05 was considered as significant. Statistical analysis was done using Stata 15 (Stata Corp, College Station, TX, USA).

## Results

A total of 379 patients were included (Tab. 1). Two patients were represented twice, given that they suffered an AMI on two distinct occasions; in these only one event (the first) was considered for the analysis. Overall, 40.9% of patients had a reduced EF upon discharge. Significant differences between patients with a reduced and preserved EF were present in the Killip classification, prior CAD, number of sessions and prescription of ticagrelor, anti-coagulants, spironolactone, diuretics, nitrates and insulin (Tab. 1).

**Table 1:** Study population characteristics

	All patients (*n* = 379)	Preserved EF (*n* = 224)	Reduced EF (*n* = 155)	*p*-value^a^
*Age (years)*	58.8 ± 10.6	58.3 ± 10.4	59.6 ± 10.9	0.259
*Male sex*	307 (81%)	181 (81%)	126 (81%)	0.905
*STEMI*	255 (67%)	146 (65%)	109 (70%)	0.294
*Revascularisation*	335 (88%)	204 (91%)	131 (85%)	0.050
*Killip classification*				
1	309 (82%)	205 (92%)	104 (68%)	<0.001
2	50 (13%)	14 (6%)	36 (23%)	
3	11 (3%)	3 (1%)	8 (5%)	
4	7 (2%)	1 (1%)	6 (4%)	
*History of CAD*	60 (16%)	23 (10%)	37 (24%)	<0.001
*Arterial hypertension*	203 (54%)	118 (53%)	85 (55%)	0.678
*Dyslipidaemia*	233 (61%)	138 (62%)	95 (61%)	0.950
*Diabetes mellitus*	100 (26%)	52 (23%)	48 (31%)	0.092
*Smoking status*				
Current smoker	173 (46%)	111 (50%)	62 (40%)	
Former smoker	74 (20%)	40 (18%)	34 (22%)	0.183
*Body mass index*	26.7 ± 3.5	26.7 ± 3.2	26.7 ± 3.8	0.920
*Ejection fraction (%)*	52 (44–56)	56 (53–58)	42 (36–45)	<0.001
*Medication at discharge*				
Acetylsalicylic acid	376 (99%)	223 (100%)	153 (99%)	0.362
Clopidogrel	226 (60%)	126 (56%)	100 (65%)	0.107
Ticagrelor	145 (38%)	95 (42%)	50 (32%)	0.046
Anti-coagulants	27 (7%)	8 (4%)	19 (12%)	0.001
ACEi/ARA	365 (96%)	216 (96%)	149 (96%)	0.879
BB	353 (93%)	206 (92%)	147 (95%)	0.276
Spironolactone	50 (13%)	0 (0%)	50 (32%)	<0.001
Diuretics	70 (18%)	15 (7%)	55 (35%)	<0.001
CCB	33 (9%)	22 (10%)	11 (7%)	0.355
Nitrates	46 (12%)	16 (7%)	30 (19%)	<0.001
Nicorandil	7 (2%)	2 (1%)	5 (3%)	0.097
Ivabradine	2 (1%)	0 (0%)	2 (1%)	0.088
Anti-diabetic agents^b^	87 (23%)	45 (20%)	42 (27%)	0.111
Insulin	15 (4%)	5 (2%)	10 (6%)	0.038
Statins	377 (99%)	223 (100%)	154 (99%)	0.793
*Number of EBCR sessions*	22 (16–25)	22 (16–24)	24 (17–27)	0.038

*ACE* angiotensin-converting enzyme inhibitors, *ARA* angiotensin II receptor blockers, *BB* beta-blockers, *CAD* coronary artery disease, *CCB* calcium-channel blockers, *EBCR* exercise-based cardiac rehabilitation, *EF* ejection fraction, *n* number of subjects, *STEMI* ST-segment elevation acute myocardial infarction

^a^ Comparison between preserved and reduced EF

^b^ Excluding insulin

Patients completed a median of 22 EBCR sessions (IQR 16-25). After the programme, pVO_2_ increased significantly (22.82 ± 6.04 vs 24.18 ± 6.31 ml/kg/min,—all patients; 24.23 ± 5.98 vs 25.16 ± 6.18 ml/kg/min, preserved EF group; 20.79 ± 5.56 vs 22.76 ± 6.25 ml/kg/min, reduced EF group; *p* < 0.001 for all comparisons), as did CPET duration [542 (480–660) vs 661 s (540–780), all patients; 600 (480–720) vs 717 s (600–780), preserved EF group; 540 (420–600) vs 660 s (484–769), reduced EF group; *p* < 0.001 for all comparisons]. There were no differences in the RER.

The reduced EF group had a lower pVO_2_ and completed a shorter CPET (Tab. 2). Those with a baseline reduced EF, however, presented a significantly higher increase in pVO_2_ and CPET duration. There were no differences between groups in the RER at the beginning of the programme, whereas at the end values differed (*p* = 0.049; Tab. 2).

**Table 2:** Comparison between functional parameters among preserved and reduced ejection fraction groups at different stages of the exercise-based cardiac rehabilitation program

	CPET1 (preserved EF)	CPET1 (reduced EF)	*p*-value
Peak VO_2_ (ml/kg/min)	24.23 ± 5.98	20.79 ± 5.56	<0.001
Percent predicted peak VO_2_	83.76 ± 15.75	72.77 ± 16.34	<0.001
RER	1.08 ± 0.11	1.06 ± 0.15	0.112
Duration (s)	600 (480–720)	540 (420–600)	<0.001
	*CPET2* *(preserved EF)*	*CPET2* *(reduced EF)*	*p-value*
Peak VO_2_ (ml/kg/min)	25.16 ± 6.18	22.76 ± 6.25	<0.001
Percent predicted peak VO_2_	86.94 ± 15.89	79.37 ± 17.67	<0.001
RER	1.09 ± 0.11	1.07 ± 0.11	0.049
Duration (s)	717 (600–780)	660 (484–769)	0.009
	*Delta* *(preserved EF)*	*Delta* *(reduced EF)*	*p-value*
Peak VO_2_ (ml/kg/min)	0.94 ± 2.87	1.97 ± 3.07	0.001
Percent predicted peak VO_2_	3.18 ± 10.01	6.59 ± 10.46	0.002
RER	0.01 ± 0.09	0.01 ± 0.13	0.936
Duration (s)	100 (60–120)	120 (60–180)	0.007

*CPET1* cardiopulmonary exercise test at the beginning of the EBCR program, *CPET2* cardiopulmonary exercise test at the end of the EBCR program, *EF* ejection fraction, *RER* respiratory exchange ratio

In the linear regression analysis, a baseline reduced EF was associated with a higher variation (delta) in pVO_2_ irrespective of age, gender, prior CAD, number of sessions, Killip class, arterial hypertension, dyslipidaemia, diabetes mellitus and smoking status (Tab. 3). This was maintained after inclusion in the model of the baseline pVO_2_ (*p* = 0.014).

**Table 3:** Multivariable linear regression analyses for the evaluation of baseline ejection fraction as a predictor of the change in peak VO_2_ after an exercise-based cardiac rehabilitation programme

Variables	β-Coefficient	Standard error	*p*-value
Age	−0.05	0.02	0.002
Sex	−0.11	0.40	0.784
Arterial hypertension	−0.50	0.33	0.132
Diabetes mellitus	0.25	0.36	0.491
Dyslipidaemia	0.94	0.34	0.005
Smoking status	−0.31	0.20	0.124
Previous history of CAD	−0.11	0.46	0.809
Number of EBCR sessions	−0.01	0.02	0.665
Killip classification	−0.29	0.27	0.296
Reduced ejection fraction	1.24	0.33	<0.001

*CAD* coronary artery disease, *EBCR* exercise-based cardiac rehabilitation

## Discussion

In the present study, we found significant improvements in pVO_2_ and CPET duration after an EBCR programme, irrespective of the baseline EF. Although those patients with a reduced EF presented worse functional capacity (as assessed by CPET), they benefitted more from this programme (delta pVO_2_ 1.97 ± 3.07 vs 0.94 ± 2.87, *p* = 0.001).

The benefit in our patient cohort, and specifically its effect among those with a reduced EF, adds to the current evidence-base for EBCR [1, 8, 25, 26]. Given that individuals with a reduced EF present a worse prognosis after an AMI [3, 20], and taking into consideration the impact of the pVO_2_ in terms of CV outcomes such as mortality, we believe these results are particularly relevant [8, 16]. Of note, the effect of EBCR was maintained after adjusting for several potential confounders, including baseline pVO_2_ (which was significantly lower among patients with a reduced EF). Although at the end of the programme RER values differed, given the differences and their absolute values (Tab. 2), we believe that this fact should not limit the interpretation of the overall results. Importantly, even small changes in pVO_2_ can be associated with positive clinical outcomes, as has been previously shown [16]. Recently De Schutter et al. reported on data from patients with coronary heart disease and showed that improvements in pVO_2_ during EBCR were associated with reduced mortality, independently of EF [27]. In this observational study, a 1 ml/kg/min greater improvement in pVO_2_ was associated with a 10% reduction in all-cause mortality [27]. As such, our data highlight the notion that EBCR presents a relevant intervention in this subgroup, characterised by a higher residual risk [1, 8, 28]. Although data show that in some series these patients are referred less often for EBCR, e.g. Brown et al. reported that although not reaching significance, patients with LV systolic dysfunction were slightly less likely to be referred [18, 24], the present study reiterates the need for adequate representation, in light of the possible benefits [1, 29].

A recent study stratifying AMI survivors with a CPET showed that those with a worse pVO_2_ reaped greater benefit from an EBCR programme [11]. Although when stratifying patients according to baseline EF no difference was noted [11] (a fact possibly explained by differences in patient characteristics and functional capacity), these data emphasise the relevance of these programmes among patients with lower functional capacity.

Patients were discharged under optimal medical therapy, and most were submitted to revascularisation during hospitalisation. As stated in the guidelines, EBCR should be combined with optimal pharmacological and device-based therapy [1, 3]. Most patients were male, and mean age was 58.8 ± 10.6 years. This is in line with current data [5, 8], and Brown et al. also reported that patients referred to these programmes were more likely to be younger, males and to have presented with an ST-elevation myocardial infarction (STEMI) [24]. Interestingly, data from the Portuguese Registry on Acute Coronary Syndromes described a mean age of 66 ± 13 years, with 44% STEMI patients [30]. This discrepancy, which we suggest could be related to differences in referrals, warrants further study on the best strategies to improve participation of less-represented populations.

The programme pre-specified 24 sessions. Despite this, patients in this study completed a median of 22 sessions (IQR 16-25). Several issues have previously been highlighted concerning hindrances related to attendance [2]. Though this data was not available for the present study, it would be of interest, in order to allow its optimisation. Although this should be kept in mind, as does the significant difference in the number of sessions between groups, a reduced EF persisted as a significant and independent predictor of a higher change in pVO_2_.

### Limitations

Several caveats should be considered when interpreting these results. Firstly, this was a retrospective observational study, with no control group. However, given previous data present on a randomised control trial [7], and the multiple clinical benefits associated with EBCR [1, 5], this should not negatively affect the interpretation of the data. Secondly, we did not assess the variation in EF during and after the EBCR programme. Though we believe it would be of interest to have such data, prior studies and meta-analyses have extensively addressed this issue [7, 8, 25]. Briefly, a meta-analysis by Zhang et al. showed that EBCR could have a positive effect on EF if started early after the event, though not all studies included showed this outcome [25]. Notably, although several studies did not report an improvement in EF, there was a consistent positive effect in pVO_2_ [7, 25]. Thirdly, this study concerns AMI survivors treated at a single tertiary centre, most of whom were subjected to percutaneous coronary intervention. Therefore, generalisation to other settings should be done with caution [17]. Fourthly, in 5.8% of individuals the CPET was not performed with the modified Bruce protocol, but with a variation given the individuals’ low functional capacity. Finally, we assessed data from a CPET in order to address functional status. In addition, although advice on a healthy lifestyle is part of the overall management by the attending physician, no specific protocol was present. It would be relevant to have data related to psychological status, as well as parameters related to body composition (namely lean body mass and adiposity), in order to fully ascertain the role of ancillary interventions within the scope of EBCR [1, 2, 8].

## Conclusion

The performance of a phase II EBCR programme was associated with significant improvements in functional capacity, expressed in pVO_2_, among a contemporary cohort of AMI survivors. Patients with a preserved and those with a reduced baseline EF derived significant benefits from this programme. Those with a reduced EF derived an even greater benefit, as assessed by the greater improvement in pVO_2_ (even when corrected for baseline exercise capacity) and exercise test duration.

## Appendix

Data collected included: age; sex; history of arterial hypertension; history of dyslipidemia or prior use of anti-dyslipidemic medication (and LDLc value); history of diabetes mellitus or prior use of anti-diabetic agents (and HbA1C value); smoking status; body mass index; prior history of coronary artery disease; type of acute myocardial infarction at presentation (ST-elevation or non ST-elevation); revascularization status; Killip classification; left ventricular ejection fraction at discharge (assessed by echocardiography); medication at discharge; number of exercise-based cardiac rehabilitation sessions attended.
